# Trigger Factors in Recurrent Corneal Erosion Syndrome

**DOI:** 10.3390/jcm14248694

**Published:** 2025-12-08

**Authors:** Gyeong Min Lee, Hae Nah Gwon, Young Joo Shin

**Affiliations:** 1 Department of Ophthalmology, Hallym University Medical Center, Hallym University College of Medicine, Seoul 07441, Republic of Korea; min126554@hallym.or.kr (G.M.L.); ghn9107@naver.com (H.N.G.); 2 Hallym BioEyeTech Research Center, Hallym University College of Medicine, Seoul 07441, Republic of Korea

**Keywords:** trigger factor, recurrent corneal erosion syndrome, alcohol drinking

## Abstract

**Background/Objectives**: Recurrent corneal erosion syndrome (RCES) is a chronic, recurrent, and painful disorder characterized by repeated episodes of sudden ocular pain due to abnormalities in the corneal epithelium or basement membrane (BM). The aim of this study was to investigate the triggering factors in patients with recurrent corneal erosion syndrome. **Methods**: Medical charts of patients diagnosed with recurrent corneal erosion syndrome among those who visited the outpatient department of Kangnam Sacred Heart Hospital between 2013 and 2017 were investigated. The trigger factors and the patient’s ocular symptoms were surveyed, and the severity of symptoms was investigated using a questionnaire. Anterior segment photos and anterior segment optical coherence tomography (AS-OCT) scans were performed. **Results**: The study included a total of 40 patients, with an average age of 46.18 (±13.81) years. Of these, 17 were men and 23 were women. Twelve patients (30%) had a history of trauma. Twelve (30%) drank alcohol the day before the onset of the disease, thirteen (32.5%) had severe fatigue, eight (20%) smoked and two (5%) exercised heavily. The severity of symptoms is related to age and fatigue. AS-OCT was performed in 18 patients; 94.4% had corneal anterior stromal hyperreflectivity, 61.1% had epithelial edema, 50.0% had irregular epithelial break-up, 44.4% had intraepithelial inclusion cysts, 33.3% had intraepithelial BM, and 11.1% had undetectable epithelial BM. Blurred vision and decreased visual acuity are associated with the absence of epithelial BM. Other AS-OCT findings showed no statistically significant difference from the visual symptoms. **Conclusions**: The most common triggering factors were fatigue and alcohol drinking. The AS-OCT findings may reflect the pathogenesis and progression of RCES.

## 1. Introduction

Recurrent corneal erosion syndrome (RCES) is a chronic and relapsing disorder characterized by repeated breakdown of the corneal epithelium, leading to episodes of acute and intense ocular pain that occur suddenly and intermittently [1]. This condition arises from abnormalities in the corneal epithelium and basement membrane (BM), resulting in poor epithelial adhesion and repeated epithelial detachment [2]. Patients typically report abrupt onset of sharp pain, a sensation of tearing or foreign-body presence, excessive tearing, light sensitivity, and eyelid swelling, particularly during sleep or upon awakening [3]. While RCES can develop following corneal trauma, many cases appear without any preceding injury [3]. Because of its unpredictable recurrences, patients often experience anxiety and fear [3,4]. Identifying precipitating factors is crucial for preventing or minimizing recurrences; however, these triggers remain poorly understood. Therefore, by examining any changes or unusual circumstances that occurred in the day preceding a relapse, patients may be able to identify potential precipitating or triggering factors for RCES episodes.

RCES is typically diagnosed based on the patient’s clinical history and findings from slit-lamp biomicroscopy [1]. Characteristic slit-lamp features include epithelial defects or areas of loosely adherent corneal epithelium, sometimes presenting as map-like lines, epithelial microcysts, dots, or fingerprint-like patterns [1]. Gentle pressure on the cornea may also produce folds in areas where the epithelium is poorly attached [5]. In recent years, anterior segment optical coherence tomography (AS-OCT) has enabled detailed in vivo visualization of the corneal epithelium, providing new opportunities for morphological assessment and diagnosis [6,7]. Therefore, in this study, the trigger factors that can induce symptoms in patients with RCES were investigated.

## 2. Materials and Methods

**Figure 1:**
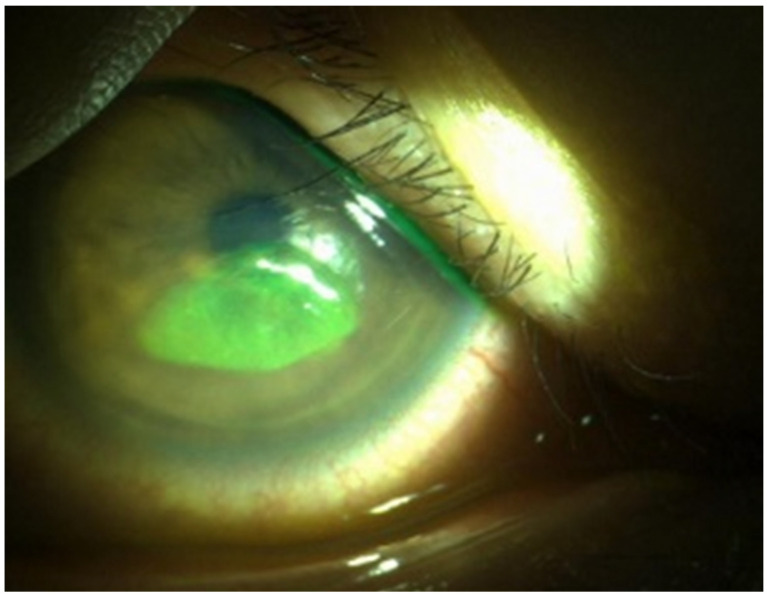
Anterior segment photography of recurrent corneal erosion.

**Figure 2:**
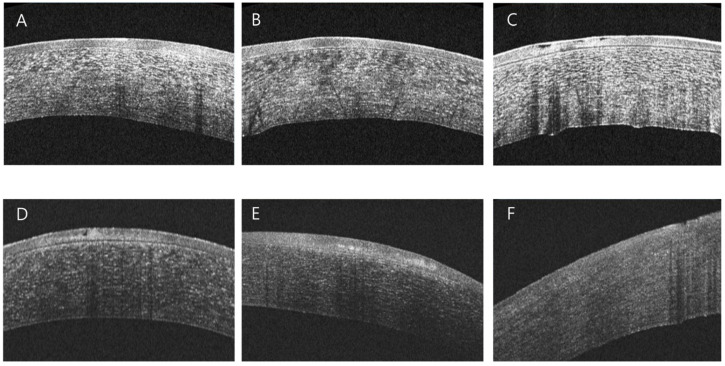
Anterior segment optical coherence tomography findings. (**A**) Anterior stromal hyperreflectivity, (**B**) corneal epithelial edema, (**C**) irregular epithelial break-up, (**D**) intraepithelial inclusion cysts, (**E**) intraepithelial basement membrane, and (**F**) undetectable epithelial basement membrane are demonstrated.

This retrospective study was performed in accordance with the tenets of the Declaration of Helsinki and was reviewed and approved by the institutional review board/ethics committee of the Hallym University Kangnam Hospital. Informed consent was not needed or waived because this study was retrospective in nature. This study enrolled patients diagnosed with RCES at the outpatient department of Gangnam Sacred Heart Hospital between 2013 and 2017. During that period, the patients underwent examinations including slit lamp microscopy and AS-OCT. RCES was diagnosed using slit-lamp biomicroscopy and topical fluorescein, characterized by irregular, loose epithelium or non-staining lesions that protrude through the tear film and usually develop at awakening [1]. A questionnaire was conducted on the possible trigger factors that occurred the day before symptom onset of the symptoms, and on the severity of symptoms. The questionnaire, presented in the Supplementary Materials, was administered to patients during a clinical visit episode. Patients retrospectively recall what they were doing the day before a relapse. Trigger factors were defined as acute lifestyle, environmental, or behavioral exposures that patients identify as immediately preceding (within 24–48 h) their recurrent corneal erosion episodes. The size of the lesion was calculated as the percentage of the lesion area with respect to the total corneal area in the anterior segment (Figure 1). AS-OCT was performed using a Cirrus HD-OCT (Carl Zeiss Meditec, Jena, Germany). Depending on the type of lesion, RCES was classified as corneal anterior stroma hyperreflectivity, epithelial edema, irregular epithelial break-up, undetectable epithelial BM, intraepithelial BM, or intraepithelial cyst, as previously described (Figure 2) [6].

To determine the trigger factor, a questionnaire test was conducted, and the participants were asked to describe what was different from the day before the onset, such as alcohol, fatigue, exercise, and sleep. In addition, the severity of symptoms was evaluated by describing the degree of symptoms, such as photosensitivity, foreign body sensation, tingling or pain, blurred vision, and decreased visual acuity, on a numerical scale from 0 to 10. The questionnaire test items included alcohol drinking, exercise status, exercise time, fatigue level, smoking status, amount smoked, contact lens wear, wearing time, sleeping time, coffee consumption, and amount taken. To minimize the likelihood that reported events were merely coincidental, we instructed patients to record activities or conditions that were different from their usual routine within the 24 h preceding symptom onset [8,9]. This approach was intended to capture meaningful deviations rather than habitual behaviors. We acknowledge that temporal association alone cannot confirm a causal relationship [10,11], and therefore the identified factors should be interpreted as potential precipitants rather than definitive triggers [8,12]. To minimize recall bias, patients were instructed to document only activities that were noticeably different from their usual routine during the 24 h preceding symptom onset, rather than recalling all activities in detail. This deviation-based reporting reduces the cognitive load associated with remembering mundane behaviors and improves the reliability of identifying meaningful pre-onset changes. Additionally, questionnaires were administered at the initial clinical visit, as close as possible to the symptomatic episode, to further limit memory decay.

### Statistics

The relationship between symptoms, lesion size, and risk factors was investigated using the Pearson correlation test, and the independent *t*-test was used to investigate whether there were any differences in symptoms according to the AS-OCT findings. Generative artificial intelligence (GenAI) tools were used to enhance the grammatical quality of the manuscript.

## 3. Results

### 3.1. Baseline Characteristics and Triggering Factors in Patients with RCES

A total of 40 patients were included in the study, with an average age of 46.18 ± 13.81 years. This study included 17 men and 23 women. Twelve patients (30%) had a history of ocular trauma. The mean lesion area accounted for 5.65 ± 4.80% of the total corneal surface. Events reported to have occurred within one day prior to symptom onset included severe fatigue in 13 patients (32.5%), alcohol consumption in 12 (30.0%), smoking in 8 (20.0%), and intense physical exercise in 2 (5.0%).

### 3.2. Association of Age and Fatigue with Symptom Severity in RCES

**Figure 3:**
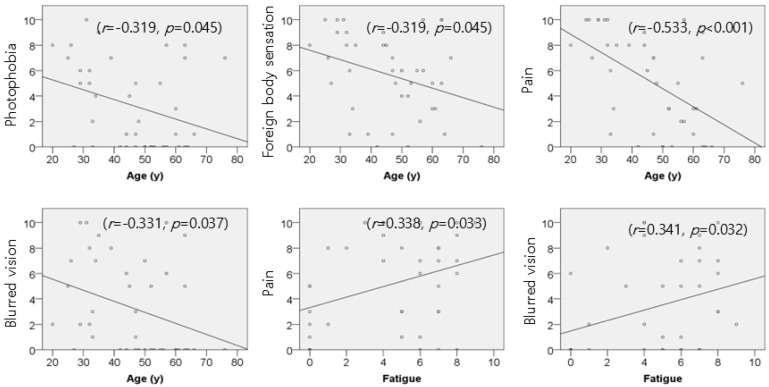
Correlation between ocular symptoms and risk factors.

The severity of clinical symptoms showed significant associations with both age and fatigue (Figure 3). Age was negatively correlated with photophobia (r = −0.319, *p* = 0.045), foreign-body sensation (r = −0.319, *p* = 0.045), pain (r = −0.533, *p* < 0.001), and blurred vision (r = −0.331, *p* = 0.037). Fatigue exhibited positive correlations with pain (r = 0.338, *p* = 0.033) and blurred vision (r = 0.341, *p* = 0.032). Lesion size did not demonstrate a significant correlation with any of the clinical parameters evaluated in this study.

### 3.3. AS-OCT-Based Analysis of Corneal Structural Changes in RCES

**Figure 4:**
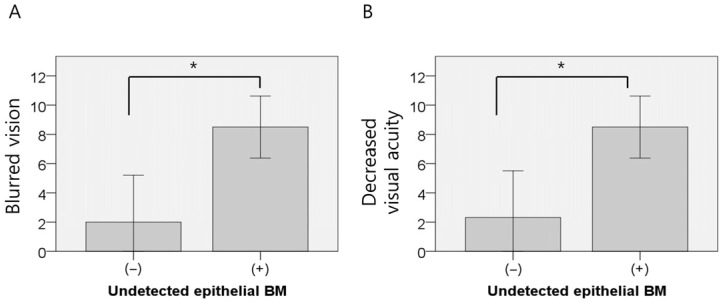
Difference in ocular symptoms according to an undetectable basement membrane. (**A**) blurred vision and undetected BM. (**B**) decreased vision and undetected BM. * *p* < 0.05.

AS-OCT was performed in 18 patients, revealing anterior stromal hyperreflectivity in 94.4%, epithelial edema in 61.1%, irregular epithelial break-up in 50.0%, intraepithelial inclusion cysts in 44.4%, intraepithelial BM in 33.3%, and an undetectable epithelial BM in 11.1% of cases. The absence of the epithelial membrane was significantly associated with blurred vision and reduced visual acuity (*p* = 0.014 and *p* = 0.018, respectively; independent *t*-test, Figure 4). No other AS-OCT findings demonstrated statistically significant associations with visual symptoms.

## 4. Discussion

RCES is a chronic and often distressing condition characterized by repeated breakdown of the corneal epithelium, resulting in recurrent ocular pain, photophobia, and tearing [1]. The conventional predisposing factors for RCES include previous ocular trauma and corneal dystrophy [13,14,15]. However, because the specific triggering events remain unclear, many patients experience anxiety regarding the unpredictable onset of symptoms [16]. In this study, any unusual event occurring within one day prior to symptom onset was defined as a potential trigger. Among the identified triggers, fatigue and alcohol consumption were the most common, each occurring with a prevalence comparable to that of a previous traumatic history—a well-established risk factor for RCES [15,16]. Alcohol is known to secrete into the tear film, alter its osmolarity, damage the corneal epithelium and induce ocular surface inflammation [17,18]. Fatigue and vigorous exercise are often associated with dehydration or mild systemic inflammation, which may compromise the epithelial barrier by promoting epithelial swelling through osmotic stress [19,20,21,22,23]. Similarly, smoking is known to contribute to subclinical systemic inflammation and tear film instability, thereby exacerbating ocular surface deterioration [24]. Impaired tear function and poor ocular surface integrity are recognized contributors to RCES pathogenesis [25].

The trigger factors identified in our study should be considered within a broader context of RCES pathophysiology. While we focused on acute lifestyle and environmental triggers, underlying chronic conditions such as meibomian gland dysfunction, dry eye disease, and systemic diseases may create a predisposing background upon which these acute triggers act [1,2,26,27]. Future studies should adopt a multifactorial approach, simultaneously assessing both chronic predisposing factors and acute triggers, to develop more personalized and comprehensive prevention strategies for RCES patients [28].

Although fatigue, alcohol consumption, and smoking are common behaviors, we attempted to improve specificity by asking patients to report only activities that differed from their usual routine during the 24 h before symptom onset [8,9]. This within-patient approach reduces the likelihood that the reported factors simply reflect background habits [10,11]. The fact that many patients independently identified similar deviations supports a non-random pattern. While a control group would strengthen causal inference [12], the consistency [29] and temporal clustering [30] of these patient-reported changes provide reasonable justification for considering them potential precipitating factors rather than purely coincidental events.

The present study also demonstrated that the severity of clinical symptoms was significantly associated with both age and fatigue. Older patients tended to experience milder photophobia, pain, and blurred vision, whereas greater fatigue was correlated with more severe pain and visual disturbance. These results suggest that systemic and behavioral factors may modulate symptom perception or corneal sensitivity in RCES. The inverse relationship between age and symptom severity in our study may be explained by several mechanisms. Age-related reduction in corneal nerve density and sensitivity is well-documented, which could lead to diminished perception of pain and discomfort in older patients [31,32]. Additionally, older adults often demonstrate elevated pain thresholds and altered central pain processing, resulting in less severe symptom reporting [33]. Psychological factors such as adaptive coping mechanisms and different symptom reporting patterns in older individuals with greater life experience may also contribute to this finding [34]. Further studies incorporating objective measures of corneal sensitivity and nerve density assessment across different age groups would help clarify the underlying mechanisms of this age-dependent symptom variation in RCES.

Although no significant correlation was found between the identified trigger factors and AS-OCT findings, the morphological characteristics of RCES on AS-OCT were categorized according to previous reports [6]. AS-OCT reveals several distinctive features in RCES, including corneal epithelial edema, intraepithelial inclusion cysts, irregular epithelial disruption, intraepithelial BM formation, anterior stromal hyperreflectivity, and absence of a detectable epithelial BM [1,6]. Corneal epithelial abnormalities are thought to arise when tear film hypo-osmolarity causes epithelial detachment from the BM, as the epithelium adheres poorly to the underlying BM following ocular surface trauma [10]. The sequential AS-OCT findings appear to mirror the disease progression, beginning with epithelial edema, followed by intraepithelial cyst formation, and eventually leading to epithelial breakup [9]. These epithelial alterations observed on AS-OCT are consistent with previously proposed pathophysiological mechanisms [9]. Severe forms of RCES are characterized by the presence of basement membrane fragments within the epithelium or by the complete absence of the BM [9]. Cases lacking a detectable BM may represent more advanced disease or underlying basement membrane pathology [1,35]. Because BM integrity is essential for epithelial adhesion, extensive BM damage likely contributes to poor epithelial stability and the development of more severe RCES phenotypes [35].

Although AS-OCT findings showed no correlation with patient symptoms other than those related to visual function, further studies are needed to clarify whether these imaging features influence treatment strategies and prognosis. In conclusion, fatigue and alcohol consumption were identified as the most common triggering factors. While AS-OCT findings were not associated with these triggers, they may nonetheless reflect the underlying pathogenesis and disease progression of RCES. AS-OCT was performed in only a subset of patients, which limits the generalizability of the imaging findings [36]. However, the imaging patterns were most relevant to RCES pathology [6]. Although the sample size is smaller than the full cohort, the consistent findings observed across these cases provide useful insight into structural alterations associated with symptom severity [1]. We therefore interpret the AS-OCT results as exploratory but meaningful observations that complement, rather than represent, the entire dataset.

Because this study was exploratory in nature [37,38] and aimed to identify potential clinical patterns rather than establish definitive causal relationships [39], we did not apply formal corrections for multiple comparisons [40]. Instead, we interpreted the results with caution, emphasizing effect sizes and consistency across related variables rather than relying solely on statistical significance [41]. Future studies with larger sample sizes and hypothesis-driven designs will be needed to apply more rigorous adjustment methods such as false discovery rate correction.

This study has several limitations. First, the small sample size restricts the statistical power and generalizability of our findings. In our study, fatigue and alcohol appeared to be common trigger factors, with higher observed prevalence compared to trauma. However, these findings require validation in larger studies. Second, recall bias may have influenced the reporting of triggering factors, as patient-reported behaviors such as fatigue or alcohol consumption were not prospectively documented. Third, the absence of a control or comparison group limits our ability to distinguish true trigger factors from background exposures common in daily life. Last, we did not control for important comorbid conditions that may influence epithelial stability or recurrence risk, including dry eye disease, ocular surface inflammation, and systemic metabolic disorders.

## 5. Conclusions

Fatigue and alcohol consumption are the most common triggering factors for RCES, showing a prevalence comparable to that of trauma, a well-known predisposing factor. Although AS-OCT findings were not correlated with these triggers or with most patient symptoms, they provide valuable insights into the structural changes and disease progression of RCES. The morphological alterations observed on AS-OCT—such as epithelial edema, cyst formation, and basement membrane disruption—likely reflect the underlying pathophysiology.

## 6. Patents

The authors declare that no patents are associated with this work.

## Data Availability

The data supporting the findings of this study are available from the corresponding author upon reasonable request.

## Supplementary Materials

The following supporting information can be downloaded at: https://www.mdpi.com/article/10.3390/jcm14248694/s1, Questionnaire for recurrent corneal erosion.

## Abbreviations

The following abbreviations are used in this manuscript:

AS-OCT	Anterior Segment Optical Coherence Tomography
BM	Basement Membrane
RCES	Recurrent Corneal Erosion Syndrome
