# Supplementary material for: Trigger Factors in Recurrent Corneal Erosion Syndrome
Source: J Clin Med. 2025 Dec 8;14(24):8694. doi: 10.3390/jcm14248694 (PMC12733406; doi:10.3390/jcm14248694)
Supplement: Supplementary file 1 [file jcm-14-08694-s001.zip › jcm-3981924-supplementary.pdf]

<Questionnaire for recurrent corneal erosion>

Age/Sex

Height (cm)/Weight (Kg)

1. Trigger factor

   Which of the following did you do the day (24h) before the onset of illness?

(1) Alcohol

   None, <1/2 bottle of Soju, 1/2~1 bottle of Soju, 1~2 bottle of Soju, >2 bottle of Soju

(2) Exercise

   None, < 30min, 30min~1h, 1h~2h, >2h

(3) Fatigue (0: best, 10: worst)

0	1	2	3	4	5	6	7	8	9	10

(4) Contact lens

   None, < 6h, 6h~12h, >12h, during sleep

(5) Sleep

   < 3h, 3h~6h, 6h~9h, >9h

(6) Coffee

   None, 1 cup, 2 cup, >2cup

2. Risk factor

(1) Diabetes mellitus: No, Yes (Duration of illness: , Management:)

(2) Trauma: No, Yes (Year of trauma)

(3) Genetic: No, Yes ( )

3. Symptoms (0: Best, 10: Worst)

(1) It's difficult to look at the light.

0	1	2	3	4	5	6	7	8	9	10

(2) Foreign body sensation

0	1	2	3	4	5	6	7	8	9	10

(3) sore or painful eye

0	1	2	3	4	5	6	7	8	9	10

(4) Objects appear to be spread out.

0	1	2	3	4	5	6	7	8	9	10

(5) I think my eyesight is deteriorating.

0	1	2	3	4	5	6	7	8	9	10
